# YAP1 Recruits c-Abl to Protect Angiomotin-Like 1 from Nedd4-Mediated Degradation

**DOI:** 10.1371/journal.pone.0035735

**Published:** 2012-04-27

**Authors:** Kassiani Skouloudaki, Gerd Walz

**Affiliations:** 1 Renal Division, University Hospital Freiburg, Freiburg, Germany; 2 Center for Biological Signaling Studies (bioss), Freiburg, Germany; Vanderbilt University Medical Center, United States of America

## Abstract

**Background:**

Tissue development and organ growth require constant remodeling of cell-cell contacts formed between epithelial cells. The Hippo signaling cascade curtails organ growth by excluding the transcriptional co-activator Yes Associated Protein 1 (YAP1) from the nucleus. Angiomotin family members recruit YAP1 to tight junctions [1], but whether YAP1 plays a specific role outside of the nucleus is currently unknown.

**Methodology/Principal Findings:**

The present study demonstrates that the E3 ubiquitin ligase Nedd4.2 targets Angiomotin-like 1 (AMOTL1), a family member that promotes the formation of epithelial tight junctions, for ubiquitin-dependent degradation. Unexpectedly, YAP1 antagonizes the function of Nedd4.2, and protects AMOTL1 against Nedd4.2-mediated degradation. YAP1 recruits c-Abl, a tyrosine kinase that binds and phosphorylates Nedd4.2 on tyrosine residues, thereby modifying its ubiquitin-ligase activity.

**Conclusions/Significance:**

Our results uncover a novel function for cytoplasmic YAP1. YAP1 recruits c-Abl to protect AMOTL1 against Nedd4.2-mediated degradation. Thus, YAP1, excluded from the nucleus, contributes to the maintenance of tight junctions.

## Introduction

Cell proliferation during development is tightly regulated to generate tissue and organs of defined size. Signaling cascades involved in the control of cell division and organ size include mTOR and the recently characterized Hippo signaling cascade [2], [3], [4], [5]. The Hippo pathway was initially established in flies; its core components such as Hippo, Salvador, Warts and Mats were subsequently identified in mammals (Mst1/2, WW45, Lats1/2 and Mob1, respectively). These four components form a kinase cascade whereby Hippo/Mst1/2 interacts with Sav/WW45 to phosphorylate and activate the protein complex of Wts/Lats1/2 and Mats/Mob1. The major target gene of the cascade is the transcriptional co-activator Yorkie (or its mammalian orthologue YAP1), which promotes cell proliferation and organ growth [6], [7]. When epithelial cells reach confluence, they exhibit contact inhibition triggered by cell-cell interactions, an event associated with nuclear exclusion and cytoplasmic retention of YAP1. This process is largely controlled by Hippo signaling, whereby phorsphorylated YAP1 by Wts/Lats1/2 is bound by the cytoplasmic 14-3-3 protein [8], [9].

It has been shown that apico-basal proteins intersect with the Hippo pathway to regulate normal tissue development [10], [11], [12], [13]. Tight junction (TJ) proteins, specifying apical and baso-lateral cell domains, can suppress proliferation by sequestering transcription factors [14]. Angiomotin like-1 (AMOTL1), a coiled-coil, PDZ-binding and glutamine rich domain containing protein [15], was recently characterized as a molecule involved in angiogenesis and cell migration [16]. AMOTL1 localizes to tight junctions (TJ), and directly interacts with MUPP1/Patj, an adaptor of the Crumbs complex [17]. AMOTL1 and AMOTL2 retain YAP1 in the cytoplasm, preventing YAP1-dependent gene activation [18]. The function of YAP1 outside the nucleus is currently unknown, although the levels of cytoplasmic YAP1 are tightly regulated via ubiquitin-dependent degradation by the E3 ligase SCF (β-TRCP) [19].

Here, we assign a novel role to cytoplasmic YAP1 in epithelial cells. We demonstrate that YAP1 binds AMOTL1 and prevents it from degradation by Nedd4.2. Nedd4.2 is a member of the NEDD4 family of E3 ligases, that contain a carboxy-terminal catalytic HECT (homolog to E6AP C-term) domain [20]. The NEDD4 family of E3-ligases regulates endocytosis and degradation of many channels, receptors, and transporters. NEDD4 E3 ligases typically contain an amino-terminal C2 domain as well as two to four WW domains that bind their substrates through recognition of (L/P)PxY motifs [21], [22], [23], [24]. Recent evidence suggests that NEDD4 proteins are also involved in tight junction assembly and the regulation of paracellular conductance in the collecting duct of the kidney [25].

We report now that Nedd4.2 targets AMOTL1 for ubiquitin-dependent degradation. YAP1 prevents AMOTL1 degradation through recruitment of the non-receptor tyrosine kinase c-Abl. Phosphorylation of Nedd4.2 by c-Abl curtails its E3 ligase activity, which results in inhibition of the ubiquitylation and degradation of AMOTL1.

## Materials and Methods

### Reagents and Plasmids

MG132 (Calbiochem) was used at a concentration of 5 µM. AMOTL1 with an N-terminal Flag tag was generated by a clone containing the human AMOTL1 cDNA (Imagenes). Full length human YAP1 and c-Abl cDNAs where cloned into the *Mlu*I and *Not*I sites of theV5-pcDNA6 vector. The human myc-Nedd4.2 C962S (dominant negative) construct was kindly provided by Dr. Pawson (Samuel Lunenfeld Research Institute, Mount Sinai Hospital, Toronto, Ontario, Canada). Dominant negative Nedd4.2 N-terminally tagged with myc was repaired to wild-type by QuickChange (QC) mutagenesis. Single and multiple amino acid substitutions were made using F9.AMOTL1 as template and QC site-directed mutagenesis with overlapping PCR primers. To create the YAP1 WW1 (^199^WQDP^202^ to ^199^AQDA^202^) and WW2 (^258^WLDP^261^ to ^258^AQDA^261^) mutants, as well as Nedd4.2 WW1 (^213^WHRP^216^ to ^213^AHRA^216^), WW2 (^385^WTRP^388^ to ^385^ATRA^388^), WW3 (^497^WEDP^500^ to ^497^AEDA^500^), WW4 (^548^WEDP^551^ to ^548^AEDA^551^), we used the wild-type templates for QC site-directed mutagenesis. The Nedd4.2 phosphorylation mutants Y71F and Y457F were generated by QC mutagenesis using the pRK5 vector. The c-Abl cDNA was re-cloned into the F9-pcDNA6 vector to obtain an N-terminal F9-tagged construct. The F9.cAbl construct was used as a template to generate the K290R kinase dead mutant, according to the manufacturer's protocol (Stratagene Cloning systems, La Jolla, CA). Ubiquitin was cloned into an HA tagged pMT123 vector into the *Not*I and *Eco*RI sites.

### Short Hairpin RNA (shRNA)

The pSUPER YAP1 human RNA interference (RNAi) designed for the target sequence 5′-ccagagaatcagtcagaga-3′ and the human Nedd4.2 for the target sequence 5′- ggatgagaatagagaacttgc-3′.

### Cell culture and Transfections

Human HEK293T and MDCK cells (received from American Type Culture Collection, ATCC, Manassas, VA) were grown in Dulbecco's modified Eagle's medium containing 10% fetal bovine serum and antibiotics. A TransPEI transfection method (Eurogentec, Cologne, Germany) was used for DNA transfections of HEK293T cells. MDCK cells were transfected with plasmid DNA using Amaxa-nucleofection (Amaxa Biosystems, Cologne, Germany). The cells were used in transient-expression experiments on the second or third day.

### Immunofluorescence

MDCK cells were fixed in 4% PFA for 20 min, permeabilized in 0.5% Triton X-100, and blocked in PBS containing 2% horse serum. Immunostainings for AMOTL1 and ZO-1 were performed with a rabbit anti-AMOTL1 (1∶200) and a mouse anti-ZO-1 (1∶250) (Zymed). HEK293T cells were fixed in 4% PFA for 10 min, permeabilized in 0.5% Triton X-100 for 10 min and blocked in PBS (0.5% Tween-20) containing 2% horse serum. Immunostainings were performed using the following antibodies: goat anti-AMOTL1 (1∶100), rabbit anti-Nedd4.2 (1∶150) and mouse anti-YAP1 (1∶150).

### Immunoprecipitations and Western Blotting

Cells were washed with ice-cold PBS and lysed with lysis buffer (20 mM Tris, pH 7.5, 1% Triton X-100, 50 mM NaCl, 50 mM NaF, 15 mM Na_4_P_2_O_7_, 0.1 mM EDTA) supplemented with 1 mM NaVO_4_ and protease inhibitor mixture (Roche). After centrifugation (15,000 g,15 min, 4°C) and ultracentrifugation (100,000 g, 30 min, 4°C) cell lysates containing equal amounts of total protein were incubated for 1 h at 4°C with the anti-Flag M2 resin (Sigma) or with anti-myc antibody, followed by incubation with 50 µl of protein A-Sepharose beads for 2 h. The beads were then washed extensively with lysis buffer and bound proteins were analyzed by Western blotting with the following antibodies: M2 antibody to Flag (Sigma), mouse anti-V5 (Serotec), mouse anti-myc 9E10 (Upstate), rabbit anti-myc A-14 (Santa Cruz), mouse anti-HA 12CA5 (Roche), rabbit anti-phosphotyrosine (BD Biosciences). HEK293T lysates were blotted for the endogenous AMOTL1, YAP1 and Nedd4.2 using the following antibodies: rabbit anti-AMOTL1 (Abcam), mouse anti-YAP1 (Abnova) and rabbit anti-Nedd4.2 (Proteintech). Rabbit anti-CEP164 was used as a negative control.

### Ubiquitylation Assay

24 h after transfection, HEK 293T cells were washed with PBS and lysed with RIPA buffer (1% Triton X-100, 0.5% sodium deoxycholate, 0.1% SDS, 150 mM NaCl, 50 mM NaF, 2 mM EDTA, 13.7 mM Na_2_HPO_4_, 6.3 mM NaH_2_PO_4_). The supernatant obtained after ultracentrifugation (100,000 g, 30 min, 4°C) was used for binding to anti-Flag M2 resin for 1.5 h, washed five times and bound proteins were resolved by SDS-PAGE. The proteins were blotted using the mouse anti-HA (Roche).

### In-gel digestion

For in-gel digestion the excised gel bands were destained with 30% ACN, shrunk with 100% ACN, and dried in a Vacuum Concentrator (Concentrator 5301, Eppendorf, Hamburg, Germany). Digests with trypsin and elastase were performed overnight at 37°C in 0.05 M NH_4_HCO_3_ (pH 8). About 0.1 µg of protease was used for one gel band. Peptides were extracted from the gel slices with 95% acetonitrile and 5% formic acid and dried in a vacuum concentrator.

### Phosphopeptide enrichment

Phosphorylated peptides were enriched using TiO_2_ (Titansphere, 5 µm particle size, GL Sciences, Japan) as recently described for phosphopeptides [26]. Briefly, peptides were re-dissolved in 10 µL 50% acetonitrile, 0.1% formic acid and loaded on a TiO_2_ nano column (0.5 cm length, 125 µm i.d.) at a flow rate of 2 µL/min. After washing with 20 µL 30% acetonitrile, 2% formic acid (flow rate: 2 µL/min), phosphorylated peptides were eluted (flow rate: 2 µL/min) with 100 mM citrate pH 9.5.

### LC-MS/MS and data analysis

LC-MS/MS analysis of the eluate from the TiO_2_ column were performed on a Q-TOF mass spectrometer (Agilent 6520, Agilent Technologies) coupled to a 1200 Agilent nanoflow system via a HPLC-Chip cube ESI interface. Peptides were separated on a HPLC-Chip with an analytical column of 75 µm i.d. and 150 mm length and a 40 nL trap column, both packed with Zorbax 300SB C-18 (5 µm particle size). Peptides were elutes with a linear acetonitrile gradient with 1% per min at a flow rate of 300 nL/min (starting with 3% acetonitrile). The Q-TOF was operated in the 2Ghz extended dynamic range mode. MS/MS analyses were performed using data-dependent acquisition mode. After a MS scan (2 spectra/s), a maximum of three peptides were selected for MS/MS (2 spectra/s). Singly charged precursor ions were excluded from selection. Internal calibration was applied using two reference masses. Mascot Distiller 2.3 was used for raw data processing and for generating peak lists, essentially with standard settings for the Agilent Q-Tof. Mascot Server 2.3 was used for database searching with the following parameters: peptide mass tolerance: 20 ppm, MS/MS mass tolerance: 0.05 Da, enzyme: “trypsin” with 2 uncleaved sites allowed for trypsin, and “none” for elastase, variable modifications: Carbamidomethyl (C), Gln->pyroGlu (N-term. Q), oxidation (M), and phosphorylation (STY). For protein and peptide identification a small custom database containing the protein sequences of Nedd4.2 was used. All MS/MS spectra identified as phosphorylated were validated manually.

## Results

### YAP1 blocks ubiquitylation and degradation of AMOTL1 by Nedd4.2

**Figure 1:**
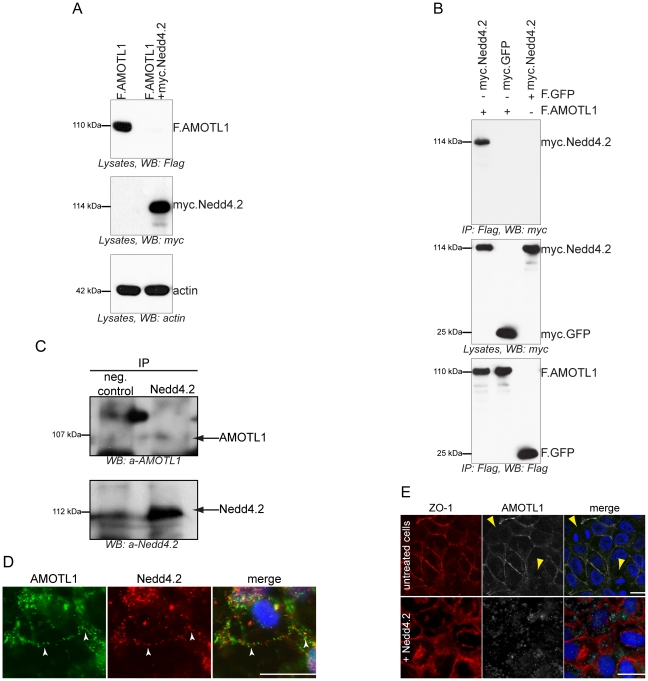
Nedd4.2 interacts with AMOTL1 and mislocalizes AMOTL1 from tight junctions. *A*, Nedd4.2 reduces AMOTL1 protein levels. Co-expression of myc-tagged Nedd4.2 (myc.Nedd4.2) decreased Flag-tagged AMOTL1 (F.AMOTL1), occasionally to very low protein levels (lane 2). *B*, AMOTL1 interacts with Nedd4.2. HEK 293T cells were transfected with constructs encoding F.AMOTL1, myc.GFP, or myc.Nedd4.2. Cell lysates were probed with anti-myc and anti-Flag antibodies. Precipitation of F.AMOTL1 immobilized myc.Nedd4.2, but not myc.GFP, while precipitated F.GFP did not bind myc.Nedd4.2. *C*, Binding of endogenous AMOTL1 to Nedd4.2 was detected with anti-AMOTL1 and anti-Nedd4.2 antibodies, respectively. CEP164 was used as a negative control. *D*, AMOTL1 co-localizes mostly at the plasma membrane with Nedd4.2 in HEK293T cells. *E*, MDCK cells were transfected either with empty vector or Nedd4.2. Cells were then stained for endogenous AMOTL1 (grayscale) and the tight junction marker, ZO-1 (red). AMOTL1 is localized to tight junctions together, with ZO-1. Note that in dividing cells (yellow arrowheads) less ZO-1 as well as AMOTL1 are observed at junctions. When Nedd4.2 is added, endogenous ZO-1 as well as AMOTL1 are mislocalized from tight junctions and appear mostly in the cytoplasm. Scale bars represent 20 µm.

**Figure 2:**
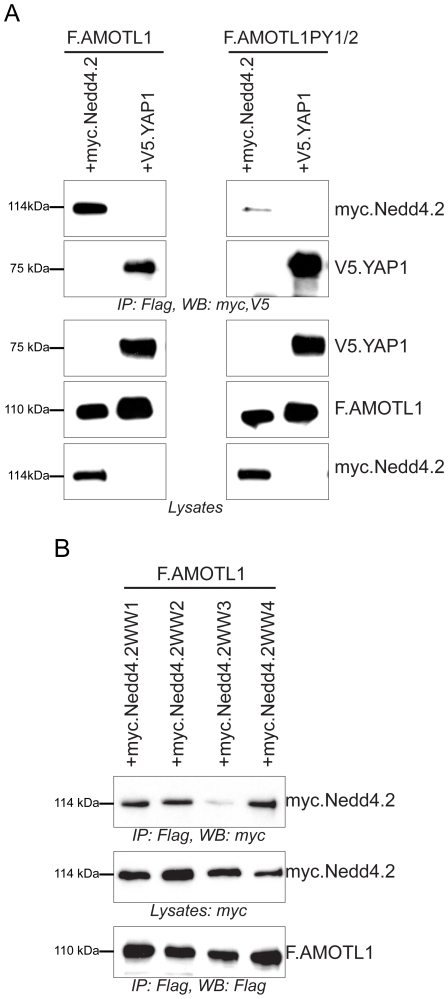
The WW3 domain of Nedd4.2 interacts with AMOTL1. *A*, The two PPxY motifs of AMOTL1 (PY1: PPEY, amino acid 310–313, and PY2: PPEY, amino acid 367–370) are important for the interaction with Nedd4.2. Precipitation of wild type F.AMOTL1 immobilized both myc.Nedd4.2 and V5.YAP1 (left panel), while precipitation of mutant F.AMOTL1PY1/2, lacking both PPxY motifs, immobilized V5.YAP1, but only small amounts of myc.Nedd4.2 (right panel). *B*, The WW1, WW2, WW3 and WW4 domains of Nedd4.2 were mutated, and co-expressed with Flag-tagged AMOTL1. Only the interaction between the myc.Nedd4.2 WW3 and AMOTL1 was decreased, indicating that this domain interacts with AMOTL1.

**Figure 3:**
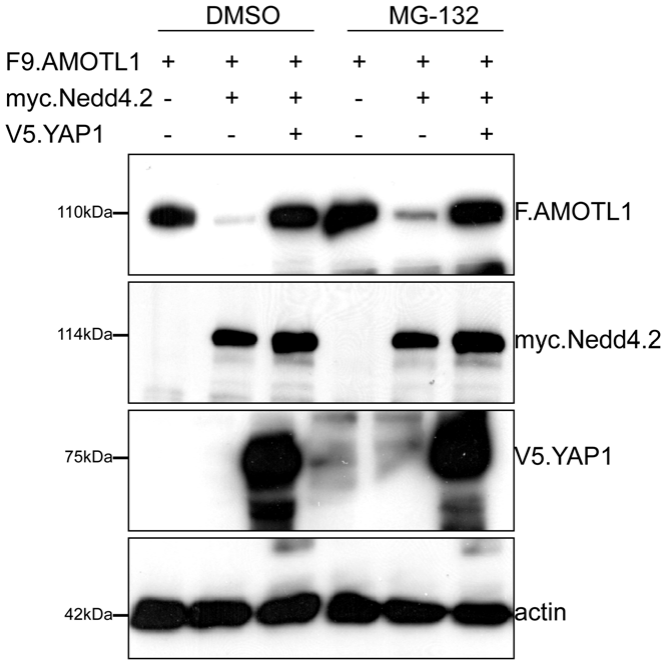
Nedd4.2 targets AMOTL1 for proteasomal degradation. HEK 293T cells were transfected with AMOTL1, Nedd4.2 and YAP1, as indicated. Cells were treated with 5 µM MG132 for 120 min. Lysates were analyzed by Western blotting. AMOTL1, Nedd4.2 and YAP1 were detected with anti-Flag, anti-myc and anti-V5 antibodies, respectively. Actin was used as a loading control.

**Figure 4:**
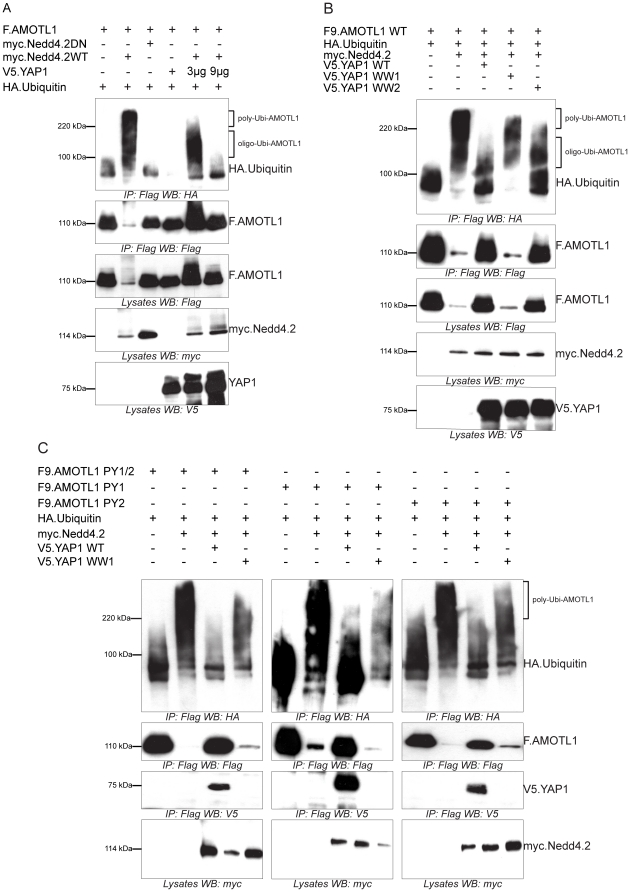
YAP1 inhibits AMOTL1 degradation via its first WW domain. *A*, Nedd4.2 facilitates the ubiquitylation of AMOTL1. HEK293T cells were transfected with AMOTL1, Nedd4.2 and YAP1, as indicated. AMOTL1 was precipitated and probed for incorporated HA-tagged ubiquitin. AMOTL1 levels were determined by re-probing the blot with anti-Flag antibody. A catalytically inactive (DN) Nedd4.2 was used as a control for ligase activity. *B*, Wild type YAP1 and the YAP1WW2 mutant, but not the YAP1WW1 mutant, reduce ubiquitylation of AMOTL1. HA-tagged ubiquitin (HA.Ubiquitin) and expression vectors, as indicated, were co-expressed in HEK293T cells. AMOTL1 was precipitated by M2 sepharose beads, and then probed with antibody to HA to identify ubiquitylated AMOTL1. *C*, YAP1 protects both single and double AMOTL1 PPxY mutants against Nedd4.2-mediated ubiquitylation. Either of AMOTL1^310–313A^ (F.AMOTL1PY1), AMOTL1^367–370A^ (F.AMOTL1PY2) or AMOTL1^310–313A/367–370A^ (F.AMOTL1PY1/2) were co-expressed with different combinations of HA.Ubiquitin, myc.Nedd4.2, V5.YAP1 and V5.YAP1 WW1, as indicated. Cell extracts were precipitated with M2 beads and blotted with anti-HA antibody. In blots for ubiquitin, brackets indicate molecular weights that correspond to poly- or oligo-ubiquitylation of AMOTL1. Poly-ubiquitylation results in complexes with molecular weights above 220 kDa which signal degradation of the protein by the 26S proteasome. Complexes between 100 kDa and 220 kDa indicate oligo-ubiquitylated peptides that are generally not degraded by the 26S proteasome.

**Figure 5:**
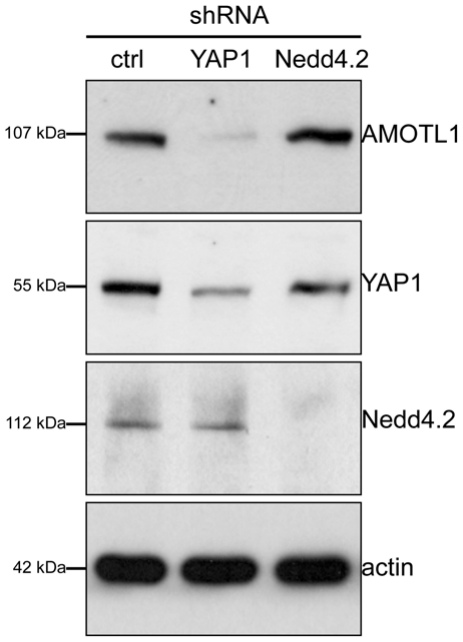
Depletion of endogenous YAP1 or Nedd4.2 affects endogenous AMOTL1 levels. HEK293T cells were transiently transfected with either YAP1 or Nedd4.2 shRNA. Even a small reduction of YAP1 results in almost complete absence of AMOTL1. However depletion of Nedd4.2 maintains AMOTL1 levels. Actin was used as a loading control.

Co-expression of AMOTL1 with Nedd4.2 resulted in a drastic reduction of AMOTL1 protein levels in HEK 293T cells; in some experiments AMOTL1 became virtually undetectable (Fig. 1A). Co-immunoprecipitation experiments revealed that the two proteins form a complex (Fig. 1B). Importantly, we could detect weak binding (Fig. 1C) and membrane co-localization of the endogenous Nedd4.2 and AMOTL1 proteins in HEK293T cells (Fig. 1D). Immunofluorescence experiments in MDCK cells showed that the endogenous AMOTL1 protein colocalises with ZO-1, a tight junction marker (Fig. 1E), but both markers are absent in dividing cells (yellow arrowheads in Fig. 1E). However, the presence of Nedd4.2 reduces the levels of AMOTL1, which appears less localized to tight junctions and is mostly cytoplasmic. In this case, ZO-1 begins to appear more diffuse and its presence at tight junctions is less pronounced (Fig. 1E). These two findings suggest that the absence of AMOTL1 tends to destabilize tight junctions. The cytoplasmic region of AMOTL1 contains two conventional WW domain binding PPxY (PY) motifs at amino acid positions 310–313 and 367–370 and one unconventional LPxY motif at 188–191 (Fig. S1). Since the WW domains of Nedd4.2 (Fig. S1) recognize PPxY motifs, we asked whether mutation of the two AMOTL1 PPxY motifs affected the interaction with Nedd4.2. Mutation of single AMOTL1 PPxY motifs to alanines had no detectable effect (data not shown), whereas substitution of both motifs (AMOTL1^310–313A/367–370A^) strongly decreased the interaction with Nedd4.2 (Fig. 2A). However, in this experiment, mutation of the two PPxY motifs of AMOTL1 did not affect the interaction with YAP1, suggesting that the PPxY mutations did not generally abolish protein-protein interactions (Fig. 2A). Site-directed mutagenesis of each of the four Nedd4.2 WW domains revealed that the third WW domain (WW3) of Nedd4.2 mediates the interaction with AMOTL1 (Fig. 2B). Based on these findings, we assumed that the E3 ubiquitin ligase Nedd4.2 targets AMOTL1 to the proteasome. To confirm this hypothesis, we probed whether inhibition of proteasomal degradation with MG132 leads to the accumulation of AMOTL1. Indeed, MG132 treatment caused partial accumulation of AMOTL1 in the presence of Nedd4.2 (Fig. 3). Furthermore, *in vivo* assays revealed that AMOTL1 was strongly ubiquitylated in the presence of wild-type Nedd4.2, but not in the presence of the dominant-negative (DN) mutant of Nedd4.2 (Fig. 4A). YAP1 protected AMOTL1 against Nedd4.2-mediated protein turnover in a dose-dependent manner (Fig. S2) and antagonized the ubiquitylation of AMOTL1 mediated by Nedd4.2 (Fig. 4A), suggesting that AMOTL1 recruits YAP1 to escape ubiquitin-dependent degradation. Importantly, we found that the same functional rules govern the relationship among endogenous AMOTL1, YAP1 and Nedd4.2. For this, we used shRNA to knock down YAP1 or Nedd4.2 in HEK293T cells that strongly express AMOTL1 [27]. We found that even partial depletion of YAP1 effectively reduces the amount of endogenous AMOTL1, whereas knockdown of endogenous Nedd4.2 maintains AMOTL1 protein levels (Fig. 5).

### YAP1, via its WW1 domain, binds and protects AMOTL1

We then asked how YAP1 protects AMOTL1. First, we corroborated our observation that overexpressed AMOTL1 and YAP1 interact physically (Fig. 2A) by examining their endogenous interaction. We found that YAP1 strongly precipitates AMOTL1 (Fig. S3A) and that the two proteins co-localize mostly at the plasma membrane (Fig. S3B). Like Nedd4.2, YAP1 contains two WW domains (Fig. S1). Therefore, we generated mutants for these two domains (WW1 and WW2). Co-expression of AMOTL1 and either of WW1- or WW2-mutant versions of YAP1 demonstrated that the interaction with AMOTL1 is mediated by the WW1 domain (Fig. S3C). In agreement with these findings, only the wild type and the WW2 mutant variant of YAP1 (which has an intact WW1 domain), prevented AMOTL1 from Nedd4.2-mediated degradation (Fig. 4B). Because longer ubiquitin chains (poly-ubiquitylation) are required for targeting to the 26S proteasome, complexes migrating higher indicate strong degradation, whereas complexes migrating lower (i.e. with shorter ubiquitin chains, oligo-ubiquitylation) efficiently avoid being recognized and degraded [28].

### The N-terminal LPTY and PPEY binding motifs of AMOTL1 are important for binding to YAP1

**Figure 6:**
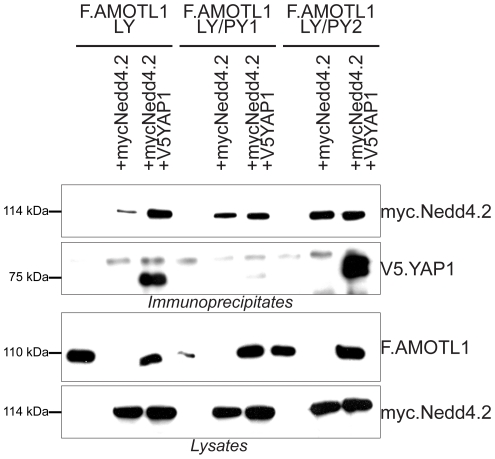
YAP1-AMOTL1 binding requires the AMOTL1 motifs LPTY and the PPEY. Either of Flag-tagged AMOTL1^188–191A^ (LY: LPTY, amino acids 188–191), AMOTL1 ^188–191A/310–313A^ (LY/PY1: LPTY, amino acids 188–191 and PPEY, amino acids 310–313) or AMOTL1 ^188–191A/367–370A^ (LY/PY2: LPTY, amino acids 188–191 and PPEY, amino acids 367–370) mutants were used to precipitate YAP1. Immunoblotting was carried out using the indicated antibodies.

WW domain-containing group I proteins such as YAP1 bind with high affinity to proline-rich PPxY (PY) motifs [29], but also recognize LPxY (LY) motifs [30], [31], [32]. For example, the group I WW domain-containing protein MAGI-1 binds the LPTY motif of AMOTL2 [33]. To identify the WW-binding motifs that are recognized by YAP1, we replaced the two PY and the single LY motifs of AMOTL1 by alanines. YAP1 precipitated the single and double AMOTL1 PY mutants (data not shown) and protected them from Nedd4.2-mediated ubiquitylation (Fig. 4C), suggesting that these two motifs play only a minor role in the AMOTL1-YAP1 complex. However, the combined LY/PY1 mutation dramatically decreased the interaction with YAP1 (Fig. 6). These findings demonstrate that AMOTL1 PPEY^310–313^ and LPTY^188–191^ are jointly required for YAP1 binding.

### Phosphorylation of Nedd4.2 by c-Abl inhibits ubiquitylation of AMOTL1

**Figure 7:**
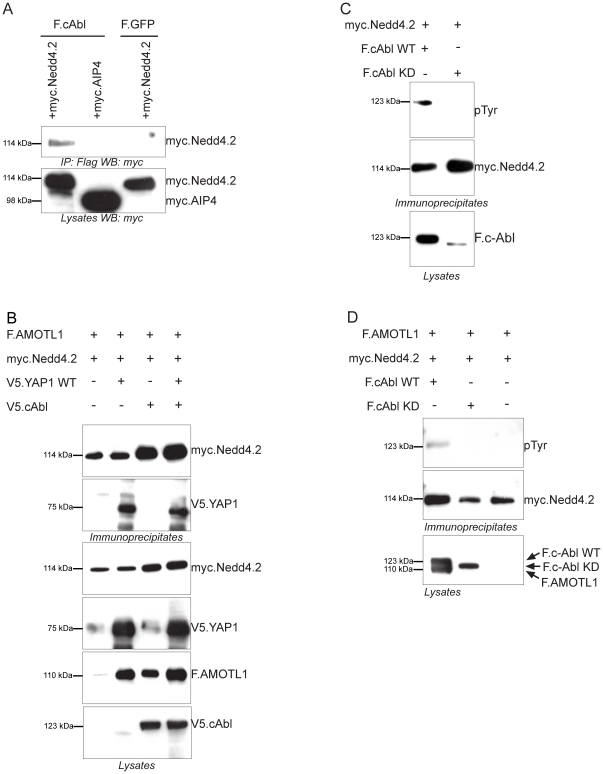
c-Abl phosphorylates Nedd4.2 which forms a triple complex with AMOTL1 and YAP1. *A*, Flag-tagged c-Abl (F.cAbl), immobilized on M2 sepharose beads, precipitates Nedd4.2. Immunoprecipitates were blotted with anti-myc antibody. AIP4, E3 ligase was used as a negative control. *B*, Protein-protein interactions among AMOTL1, Nedd4.2 and YAP1. HEK293T cells transfected with Flag-tagged AMOTL1, myc.Nedd4.2, V5.YAP1 and V5.cAbl plasmids were lysed and immunoprecipitation was performed using M2 beads. Precipitates were blotted with the antibodies indicated. Increased levels of AMOTL1 were detected in the presence of YAP1 and c-Abl. *C*, HEK293T cells were co-transfected with myc.Nedd4.2 and either wild type or kinase-dead (KD) c-Abl. Immunoprecipitation was performed with anti-myc antibody. Samples were blotted with anti-phosphotyrosine antibody to test for Nedd4.2 phosphorylation. *D*, Tyrosine phosphorylation of Nedd4.2 by c-Abl inhibits AMOTL1 degradation. Flag-tagged AMOTL1 (F.AMOTL1) and myc-tagged Nedd4.2 (myc.Nedd4.2) were co-expressed with either wild type or kinase-dead Flag-tagged c-Abl (F.cAbl). Precipitates, immobilized with anti-myc antibody, were probed with anti-phosphotyrosine and with anti-myc antibody show equal amounts of Nedd4.2; staining with anti-Flag revealed AMOTL1 protein levels.

**Figure 8:**
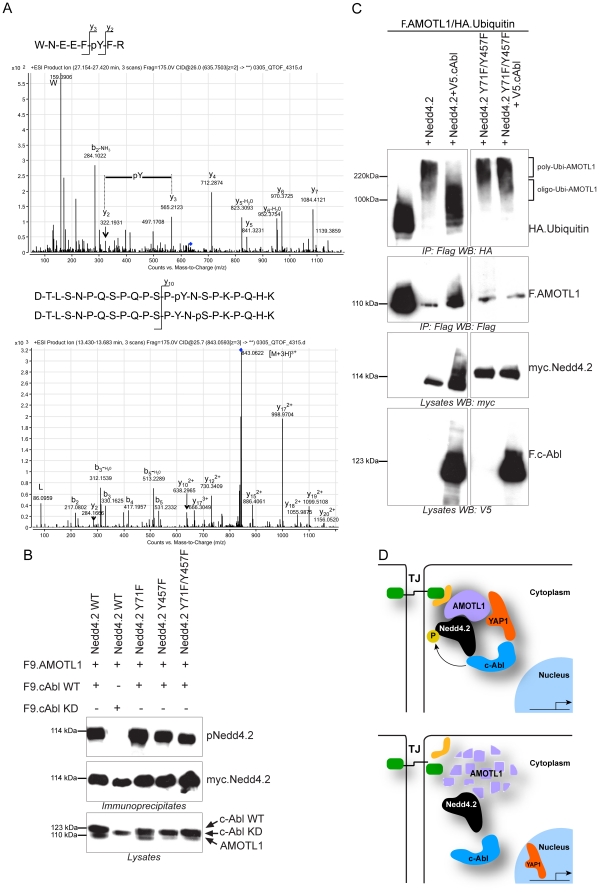
Phosphorylation of Nedd4.2 by c-Abl at tyrosine 71 and 457 inhibits its E3 ligase activity. *A*, Myc-Nedd4.2 proteins purified from HEK293T cells, co-expressing either wild type or kinase-dead (KD) c-Abl, were analyzed by nanoLC-MS/MS after in-gel digestion and phosphopeptide enrichment. Two singly Y-phosphorylated peptides (Y71 and Y457) were identified. *B*, Nedd4.2 proteins with Y71F and Y457F substitutions exhibit moderately reduced levels of c-Abl-mediated tyrosine phosphorylation. HEK293T cells were transfected with plasmids encoding myc-tagged wild type (WT) Nedd4.2 or mutants (Y71F, Y457F, or Y71F/Y457F), together with Flag-tagged AMOTL1 and WT or kinase-dead c-Abl, as indicated. Nedd4.2 proteins were precipitated with anti-myc antibodies and blotted with antibodies, as indicated. c-Abl and AMOTL1 were detected in the lysates with anti-Flag antibody. *C*, c-Abl restricts the ability of Nedd4.2 to extend the poly-ubiquitin chains of AMOTL1. Flag-tagged AMOTL1 (F.AMOTL1) and HA.Ubiquitin were co-expressed with either myc.Nedd4.2 alone, or with myc.Nedd4.2 and V5.cAbl. Ubiquitylated AMOTL1 species, detected by anti-HA antibodies, were shifted from higher molecular weights (poly-Ubi-AMOTL1) above 220 kDa towards lower molecular weight complexes between 100 kDa and 220 kDa (oligo-Ubi-AMOTL1) which are no longer degraded (see text for details). Nedd4.2^Y71F/Y457F^ is resistant to c-Abl phosphorylation. While c-Abl reduced the molecular weight of ubiquitylated AMOTL1 (left panel), there was no detectable change in molecular weight of ubiquitylated AMOTL1 (right panel) when c-Abl was co-expressed with the double tyrosine Nedd4.2 (Nedd4.2^ Y71F/Y457F^) mutant. *D*, The dual functions of YAP1. Upper panel: AMOTL1 recruits cytoplasmic YAP1 and c-Abl to tight junctions to curtail the ability of Nedd4.2 to poly-ubiquitylate AMOTL1. Lower panel: In cells primed to undergo proliferation, YAP1 translocates to the nucleus. Now, Nedd4.2 poly-ubiquitylates AMOTL1 and targets it for degradation.

YAP1 binds the SH3 domain of Src kinase family members, and has been shown to interact with the non-receptor tyrosine kinase c-Abl [34], [35]. Because c-Abl phosphorylates and inactivates the E3-ligase Parkin [36], we speculated that YAP1 recruits c-Abl to phosphorylate and inhibit Nedd4.2 activity. Precipitation of c-Abl immobilized Nedd4.2, but not the E3 ubiquitin ligase AIP4 (Fig. 7A). Moreover, YAP1 does not compete with Nedd4.2 for binding to AMOTL1, but forms a multimeric protein complex with Nedd4.2 and AMOTL1 (Fig. 7B). In addition, in the absence of YAP1, c-Abl is able to protect AMOTL1 from degradation by Nedd4.2 to some extent, although the simultaneous addition of YAP1 significantly increases AMOTL1 stabilization (Fig. 7B). Monitoring overall tyrosine-phosphorylation of Nedd4.2 revealed that Nedd4.2 is phosphorylated on tyrosine residues in the presence of wild type c-Abl, but not in the presence of the kinase-dead c-Abl (c-Abl^K290R^) (Fig. 7C). Consistent with our hypothesis that tyrosine phosphorylation blocks Nedd4.2 E3 ligase activity, we observed increased AMOTL1 levels in the presence of wild-type c-Abl, but not in the presence of the kinase-dead c-Abl mutant (Fig. 7D). To identify which Nedd4.2 tyrosine residues are phosphorylated in the presence of c-Abl, we precipitated Nedd4.2 from cells expressing either wild-type c-Abl or c-Abl^K290R^, and subjected purified Nedd4.2 to mass spectrometry analysis. This approach revealed two phosphorylation sites within the peptide sequences WNEEFY*FR and DTLSNPQSPQPSPY*NSPK, corresponding to Y71 and Y457 of human Nedd4.2 (NCBI Accession NP_001138441) (Fig. 8A). To validate the significance of Y71 or Y457 phosphorylation, we replaced the two tyrosines of myc-tagged Nedd4.2 with phenylalanines. Phosphorylation of Nedd4.2^Y71F^ and Nedd4.2^Y457F^ was not reduced; moderate reduction in phosphorylation was only detected for the Nedd4.2^Y71F,Y457F^ double mutant (Fig. 8B). To test whether the two Nedd4.2 tyrosine residues are important for c-Abl-mediated inhibition of Nedd4.2 function, we assessed the ubiquitylation of AMOTL1 in the presence of c-Abl with wild type and mutant Nedd4.2^Y71F,Y457F^. Nedd4.2 promoted the formation of poly-ubiquitylated AMOTL1 species with a molecular weight above 220 kDa. The presence of c-Abl decreased the molecular weight range of ubiquitin-bound AMOTL1 between 100 and 220 kDa, consistent with the formation of AMOTL1 molecules with shorter ubiquitin chains (oligo-ubiquitylation). Nedd4.2^Y71F,Y457F^ was resistant to the action of c-Abl and polyubiquitylated AMOTL1 was produced even in the presence of c-Abl (Fig. 8C). As previously explained for Fig. 4B, longer ubiquitin chains (MW greater than 220 kDa) indicate 26S proteasome-mediated degradation, whereas shorter ubiquitin chains are not sufficient to target the protein for degradation. The above findings support the hypothesis that c-Abl mediates tyrosine phosphorylation to restrain the E3 ligase activity of Nedd4.2.

## Discussion

Recent studies have shown that different members of the AMOT protein family (AMOT, AMOTL-1 and AMOTL-2) exhibit similar function by interacting with and regulating YAP1 subcellular localization. However, the functional importance of these family members can vary between cell lines and tissues, depending on their relative expression levels [18], [37]. Like AMOT, AMOTL1 has been reported to be a tight junction-associated protein [17]. Other studies support that YAP1 adopts tight junction localization in polarized epithelial cells in an AMOT-dependent manner [37]. Therefore, the role of YAP1 in tight junction maintenance through the regulation of AMOTL1 is intriguing.

Angiomotin proteins co-evolved with the advent of vascular epithelium [38]. Since *Drosophila melanogaster* lacks continuous vasculature, functional homologs of Angiomotin proteins are absent in flies. Thus, cytoplasmic retention of Yorkie (YAP1) appears to rely predominantly on Hippo signaling in *Drosophila*. Polarized vertebrate cells expressing AMOT family members can prevent nuclear translocation of YAP1 by recruiting this transcriptional activator to tight junctions [18], [19]. Our results, however, highlight a novel and unexpected function of YAP1 at tight junctions. While Nedd4.2 targets AMOTL1 for ubiquitin-dependent degradation, YAP1 recruits c-Abl to facilitate phosphorylation and inhibition of Nedd4.2.

Our findings uncover a dual and opposing role for YAP1 in the nucleus and the cytoplasm (Fig. 8D). Polarized non-dividing cells need to maintain cell-cell contacts and epithelial integrity, which in turn requires TJ-associated AMOTL1 and therefore cytoplasmic YAP1. Cytoplasmic YAP1 in this case acts as an adaptor protein that recruits the tyrosine kinase c-Abl to associate with Nedd4.2, resulting in its phosphorylation at Y71 and Y457. This complex then modulates Nedd4.2 activity such, that the length of ubiquitin chains added on AMOTL1 is limited, protecting it from 26S proteasome degradation. On the other hand, when cells undergo proliferation, YAP1 translocates to the nucleus to associate with TEAD family transcription factors, implicated in tumor growth and metastasis [39]. Meanwhile, AMOTL1 is exposed to degradation by Nedd4.2, which leads to TJ disassembly, required in dividing cells.

## Supporting Information

Figure S1 **Domain structure of AMOTL1, YAP1 and Nedd4.2.** WW domains in AMOTL1 and Nedd4.2 protein sequence are indicated with blue rhombs. c-c, coiled coli domains; P-rich, proline rich domains; C2, C2-domain; HECTc, Homologous to the E6-AP Carboxyl Terminus domain.
(TIF)

Figure S2 **YAP1 protects AMOTL1 against Nedd4.2-mediated protein turnover.** F.AMOTL1, and myc.Nedd4.2 were co-expressed with increasing amounts of V5.YAP1 (1 µg, 3 µg and 6 µg). Western blot analysis with anti-Flag revealed increasing AMOTL1 levels despite the presence of myc.Nedd4.2. Actin was used as a loading control.
(TIF)

Figure S3 **The WW1 domain of YAP1 is required for binding and stabilization of AMOTL1.** *A*, Endogenous AMOTL1 was strongly precipitated by a mouse anti-YAP1 antibody. *B*, Immunostaining in HEK293T cells using anti-AMOTL1 and anti-YAP1 antibodies revealed that the two proteins localize to the cell membrane. *C*, YAP1 WW1 and WW2 mutants were co-expressed with AMOTL1 in HEK 293T cells. AMOTL1 was precipitated with anti-Flag. YAP1WW2, but not YAP1WW1 was immobilized by AMOTL1, and detected by anti-V5 antibody. GFP was used as a negative control. Scale bars represent 20 µm.
(TIF)
