# Supplementary figures and images for: YAP1 Recruits c-Abl to Protect Angiomotin-Like 1 from Nedd4-Mediated Degradation
Source: PLoS One. 2012 Apr 27;7(4):e35735. doi: 10.1371/journal.pone.0035735 (PMC3338797; doi:10.1371/journal.pone.0035735)

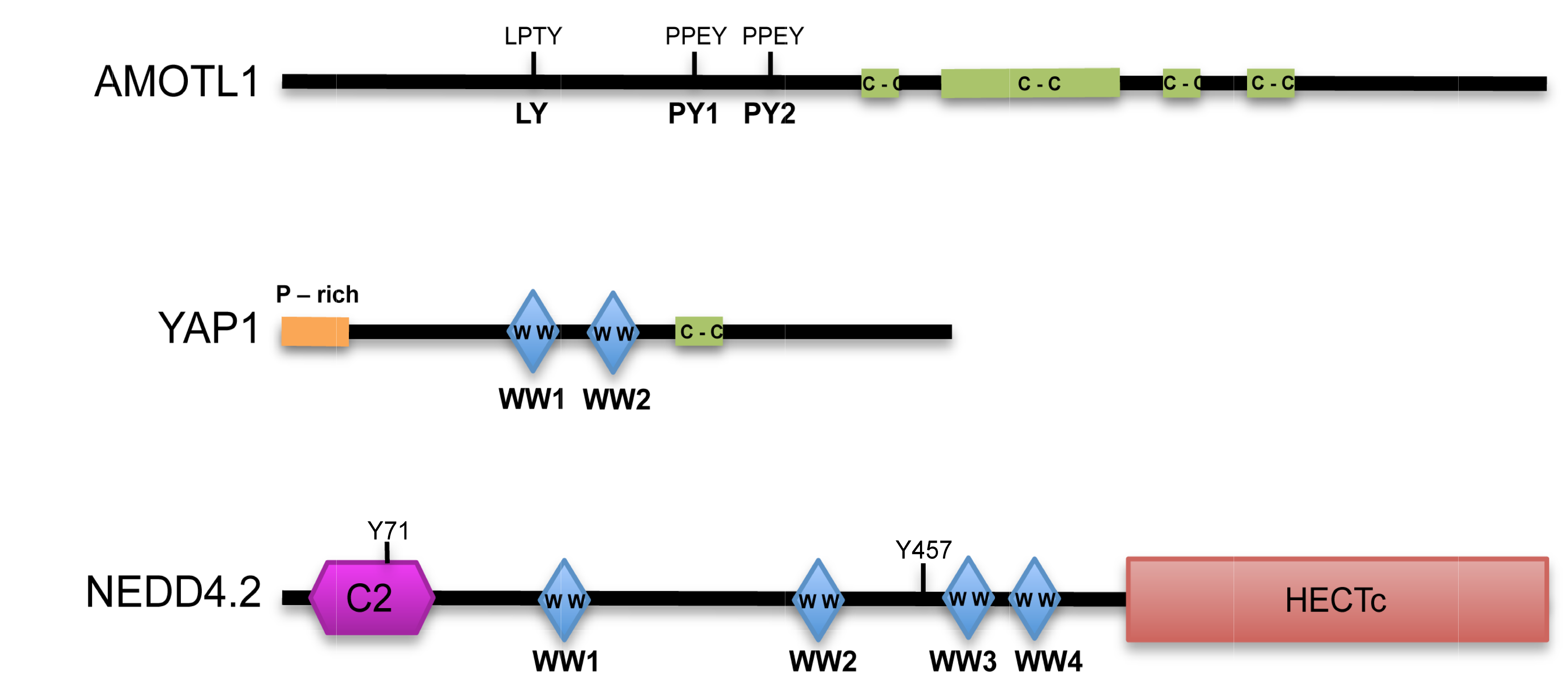

Supplement: Figure S1 — Domain structure of AMOTL1, YAP1 and Nedd4.2. WW domains in AMOTL1 and Nedd4.2 protein sequence are indicated with blue rhombs. c-c, coiled coli domains; P-rich, proline rich domains; C2, C2-domain; HECTc, Homologous to the E6-AP Carboxyl Terminus domain. (TIF) [file pone.0035735.s001.tif]

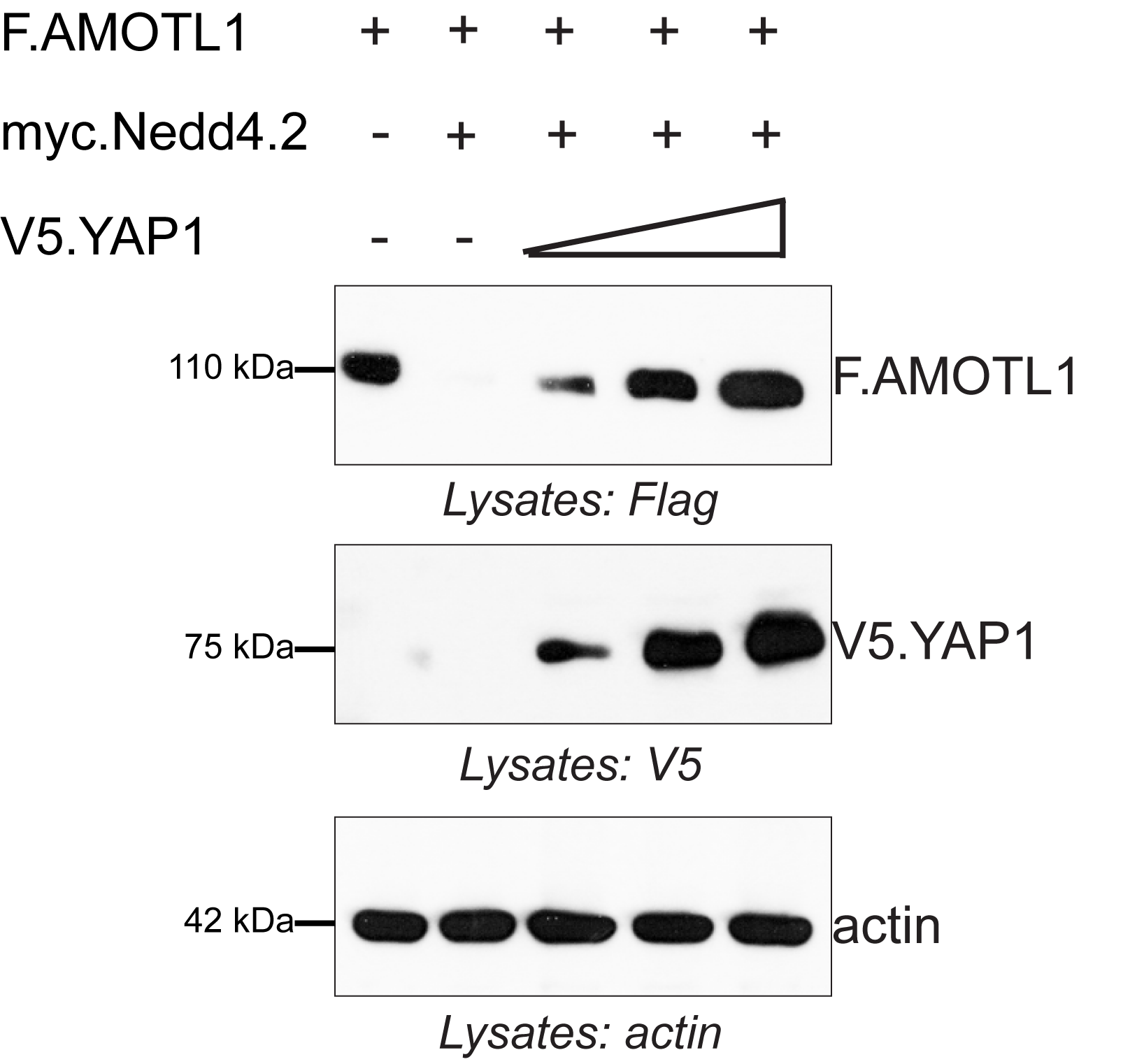

Supplement: Figure S2 — YAP1 protects AMOTL1 against Nedd4.2-mediated protein turnover. F.AMOTL1, and myc.Nedd4.2 were co-expressed with increasing amounts of V5.YAP1 (1 µg, 3 µg and 6 µg). Western blot analysis with anti-Flag revealed increasing AMOTL1 levels despite the presence of myc.Nedd4.2. Actin was used as a loading control. (TIF) [file pone.0035735.s002.tif]

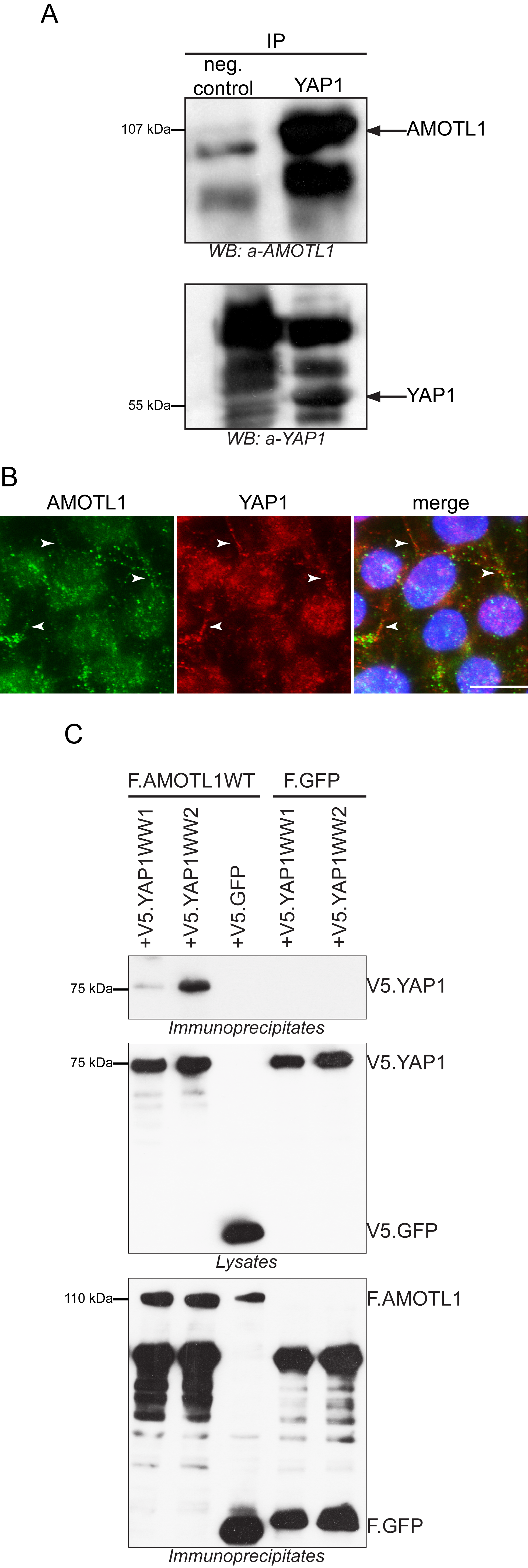

Supplement: Figure S3 — The WW1 domain of YAP1 is required for binding and stabilization of AMOTL1. A, Endogenous AMOTL1 was strongly precipitated by a mouse anti-YAP1 antibody. B, Immunostaining in HEK293T cells using anti-AMOTL1 and anti-YAP1 antibodies revealed that the two proteins localize to the cell membrane. C, YAP1 WW1 and WW2 mutants were co-expressed with AMOTL1 in HEK 293T cells. AMOTL1 was precipitated with anti-Flag. YAP1WW2, but not YAP1WW1 was immobilized by AMOTL1, and detected by anti-V5 antibody. GFP was used as a negative control. Scale bars represent 20 µm. (TIF) [file pone.0035735.s003.tif]
